# Culture Strategies for Isolation of Fastidious *Leptospira* Serovar Hardjo and Molecular Differentiation of Genotypes Hardjobovis and Hardjoprajitno

**DOI:** 10.3389/fmicb.2017.02155

**Published:** 2017-11-02

**Authors:** Roberta T. Chideroli, Daniela D. Gonçalves, Suelen A. Suphoronski, Alice F. Alfieri, Amauri A. Alfieri, Admilton G. de Oliveira, Julio C. de Freitas, Ulisses de Padua Pereira

**Affiliations:** ^1^Department of Veterinary Preventive Medicine, State University of Londrina, Londrina, Brazil; ^2^Department of Veterinary Preventive Medicine and Public Health, Paranaense University, Umuarama, Brazil; ^3^National Institute of Science and Technology for Dairy Production Chain (INCT - LEITE), Universidade Estadual de Londrina, Londrina, Brazil; ^4^Laboratory of Microbial Biotechnology, Department of Microbiology, State University of Londrina, Londrina, Brazil

**Keywords:** leptospirosis, culture medium, pyruvate sodium, superoxide dismutase, fetal bovine serum, DNA fingerprint

## Abstract

The *Leptospira* serovar Hedjo belongs to the serogroup sejroe and this serovar is the most prevalent in bovine herds worldwide. The sejroe serogroup is the most frequently detected by serology in Brazilian cattle herds suggesting that it is due serovar Hardjo. In the molecular classification, this serovar has two genotypes: Hardjobovis and Hardjoprajitno. This serovar is as considered as fastidious pathogens, and their isolation is one of the bottlenecks in leptospirosis laboratories. In addition, its molecular characterization using genomic approaches is oftentimes not simple and time-consuming. This study describes a method for isolating the two genotypes of serovar Hardjo using culture medium formulations and suggests a get-at-able molecular characterization. Ten cows naturally infected which were seropositive were selected from small dairy farms, and their urine was collected for bacterial isolation. We evaluated three modifications of liquid *Leptospira* medium culture supplemented with sodium pyruvate, superoxide dismutase enzyme and fetal bovine serum, and the isolates were characterized by molecular techniques. After isolation and adaptation in standard culture medium, the strains were subcultured for 1 week in the three modified culture media for morphologic evaluation using electronic microscopy. Strains were molecularly identified by multilocus variable-number tandem-repeat analysis (MLVA), partial sequencing and phylogenic analyses of gene *sec Y*. Combining the liquid culture medium formulations allowed growth of the *Leptospira* serovar Hardjo in three tubes. Two isolates were identified as genotype Hardjobovis, and the other as genotype Hardjoprajitno. Morphologically, compared with control media, cells in the medium supplemented with the superoxide dismutase enzyme were more elongated and showed many cells in division. The cells in the medium supplemented with fetal bovine serum were fewer and lost their spirochete morphology. This indicated that the additional supplementation with fetal bovine serum assisted in the initial growth and maintenance of the viable leptospires and the superoxide dismutase enzyme allowed them to adapt to the medium. These culture strategies allowed for the isolation and convenient molecular characterization of two genotypes of serovar Hardjo, creating new insight into the seroepidemiology of leptospirosis and its specific genotypes. It also provides new information for the immunoprophylaxis of bovine leptospirosis.

## Introduction

The genus *Leptospira* comprises a heterogeneous group of pathogenic and saprophytic species belonging to the order *Spirochaetales* (Adler and de la Peña Moctezuma, 2010). Leptospiral serovar diversity results from structural heterogeneity in the carbohydrate component of the lipopolysaccharides (de la Peña-Moctezuma et al., 1999). Many serovars are adapted for specific mammalian hosts, which harbor these microorganisms in the renal tubules and intermittently eliminate them through the urine contaminating the surrounding environment (Adler and de la Peña Moctezuma, 2010).

Serovar Hardjo is one of serovars of sejroe serogroup. In bovine herds naturally infected, the serovar Hardjo is the most prevalent (Ellis, 2015). In Brazilian cattle herds, antibodies against the sejroe serogroup are the most frequently detected by the microscopic agglutination test (MAT) (Favero et al., 2001; Figueiredo et al., 2009; Hashimoto et al., 2012; Silva et al., 2012), suggesting that it is due to the serovar Hardjo. By molecular classification, this serovar has two genotypes (Hardjobovis and Hardjoprajitno). The genotype Hardjobovis belongs to the species *Leptospira borgpetersenii* and genotype Hardjoprajitno to the species *Leptospira interrogans*.

Serological methods are limited in that they can only distinguish the serovars at the serogroup level but cannot differentiate the genotypes of the Hardjo serovar (Picardeau, 2013), which are relevant for the epidemiology of these genotypes. Serovar determination is a very laborious methodology and the use of monoclonal antibody panels for the cross-agglutinin absorption test (CAAT), has a high cost for the implementation of this methodology and may only be performed at the Royal Tropical Institute reference laboratory in the Netherlands (Faine et al., 1999). Isolating leptospiral strains is useful for molecular characterization and genotyping; however, it is time-consuming and uncertain, particularly for the more fastidious serovars such as Hardjo (Pailhoriès et al., 2015). These microorganisms are slow-growing and require a rich medium at a neutral pH, which makes it difficult to cultivate leptospires from natural sources (Johnson and Gary, 1962; Staneck et al., 1973; Bey and Johnson, 1978; Adler et al., 1986; González et al., 2006; Zacarias et al., 2008).

Serovar Hardjo is difficult to culture and with low rates of success in the attempts of isolation due to its extreme nutritional requirements (Robertson et al., 1964; Flint and Liardet, 1980; Ellis and Thiermann, 1986; Leonard et al., 1992). While ordinary culture media are adequate to recover the less fastidious leptospires, they are ineffective for isolating serovar Hardjo.

Many studies are working to improve the culture media used for isolating *Leptospira* spp. by adding components that help the bacteria grow such as sodium pyruvate, different concentrations of Tween 80, bovine serum albumin (Johnson et al., 1973; Rodríguez et al., 2002; González et al., 2006; Wuthiekanun et al., 2014), and different combinations of antibiotics that inhibit contaminants (Johnson and Rogers, 1964; Myers, 1975; Zacarias et al., 2008; Miraglia et al., 2009; Chakraborty et al., 2011). However, there are few studies regarding the effect of these supplements on the initial isolation and maintenance of the strains as well as the supplement's influence on the viability, motility, and leptospiral cell morphology.

In the last three decades, molecular methods such as pulse field gel electrophoresis (PFGE), restriction fragment length polymorphism (RFLP), Multiple Locus Sequence Typing (MLST), and multilocus variable-number tandem-repeat analysis (MLVA) were introduced to diagnosis, identification and characterization of leptospires (Thiermann et al., 1985; Herrmann et al., 1992; Perolat et al., 1993; Ralph et al., 1993; Majed et al., 2005; Ahmed et al., 2006). In particular, the MLVA method is useful, low time-consuming and has accessible costs for detecting and identifying *Leptospira* serovars (Pourcel et al., 2003), including the genotype Hardjobovis of serovar Hardjo (Chideroli et al., 2016).

This work describes different culture media compositions for isolating two genotypes of serovar Hardjo, their molecular characterization by MLVA, and their phylogeny using a partial sequence of the *secY* gene.

## Materials and methods

### Selection of animals

Cows naturally infected from three small dairy farms in the northern Parana state were monitored serologically for leptospirosis due to their history of reproductive failure. Of these animals, 10 cows that tested positive on the MAT test (titers between 100 and 1,600 for serogroup sejroe) were selected, and their urine was collected for bacterial isolation and DNA detection by PCR.

### Animal ethics and usage

The study was carried out in accordance with the recommendations of National Council for Control of Animal Experimentation (CONCEA). The protocol was approved by The Ethics Committee on Animal Use (CEUA) from State University of Londrina number CEEA - 58/06.

### Preparation of the new culture medium

Three formulations of base liquid culture media were produced to isolate and maintain the leptospires. All media contained basic ingredients and additional supplements (Table 1).

**Table 1:** Resume of the combinations of the EMJH culture media.

Culture medium^*^	**Formulation**
A	EMJH base medium + base enrichment + sodium pyruvate
B	EMJH base medium + base enrichment + sodium pyruvate + superoxide dismutase enzyme
C	EMJH base medium + fetal bovine serum + sodium pyruvate + superoxide dismutase

* *All culture media were produced both with and without antibiotics*.

Culture medium A contained *Leptospira* Medium Base culture Ellinghausen-McCullough-Johnson-Harris (EMJH) (2.56 g/L; Difco™, InterLab, BR), *Leptospira* Enrichment EMJH (100 mL/L; Difco™, InterLab, BR), and sodium pyruvate (0.1 g/L; Sigma®, USA). Medium B contained the same components as medium A with the addition of the enzyme, superoxide dismutase (25,000 U/L; Sigma®, USA). Medium C was similar to B; however, the base Enrichment EMJH (rabbit serum supplement) was changed to fetal bovine serum (100 mL/L; Gibco®, USA).

When necessary, the following antibiotics were added to the three culture media formulations: 5-fluorouracil (400 mg/L, Sigma®, USA), chloramphenicol (5 mg/L, Sigma®, USA), nalidixic acid (50 mg/L, Sigma®, USA), neomycin (10 mg/L, Sigma®, USA), and vancomycin (10 mg/L, Acros®, USA) (Zacarias et al., 2008).

### Urine collection and culture

A urine sample from each animal was obtained by perineal massage and immediately seeded in tubes containing either culture medium A, B, and C with the five antibiotics. After incubation at 28°C for 24 h, the cultures were seeded in duplicate using the same three different culture media without antibiotics. The initial tubes with antibiotics were discarded after subculturing for 24 h, and the subcultures tubes were evaluated weekly for 6 months with a dark field microscope (Olympus BX40 Model).

### Extraction and amplification of DNA for MLVA and *secY*

For genetic characterization, DNA from the leptospire cultures was extracted using the PureLink Genomic DNA Mini Kit (Invitrogen Life Technologies, Eugene, OR, USA). DNA from the leptospiral strain isolates was amplified using the Platinum PCR SuperMix Kit (Invitrogen Life Technologies, Eugene, OR, USA) according to the following conditions: 45 μL of each reaction containing SuperMix, 1 μL of each primer (10 nM), and 3 μL of DNA template (~50 ng). All products were analyzed by electrophoresis in a 2% agarose gel with ethidium bromide (0.5 g/mL) in 0.5X TBE buffer (89 mM Tris, 89 mM boric acid, and 2 mM EDTA), pH 8.4, and visualized under ultraviolet light. Molecular size was estimated by comparison with a 100-bp ladder.

### Molecular typing of the isolates

To characterize the *Leptospira* strains, two molecular techniques were used. The MLVA identified isolates with five primer pairs for the VNTR loci 4, 7, 10, LB4, and LB5 as previously described (Salaün et al., 2006). For each of the five PCRs, the VNTR loci were used as positive controls for the reference strains of *L. interrogans* serovar Canicola serogroup canicola strain Canicola Hond Utrecht IV, *L. interrogans* serovar Hardjo serogroup sejroe genotype Hardjoprajitno strain Hardjoprajitno, and *L. borgpetersenii* serovar Hardjo serogroup sejroe genotype Hardjobovis strain Sponselee. After amplification, the sequencing of *secY* was used to identify and confirm genetic species, as previously described (Ahmed et al., 2006).

The products of the *secY* gene amplification were purified with a Purelink Genomic DNA extraction kit (Invitrogen Life Technologies, Eugene, OR, USA), quantified by a Qubit™ Fluorometer (Invitrogen Life Technologies, Eugene, OR, USA), and sequenced on a ABI3500 Genetic Analyzer (Applied Biosystems, Foster City, CA, USA) using forward and reverse primers. Sequence quality was analyzed by the Phred program (http://asparagin.cenargen.embrapa.br/phph/). The consensus sequences were obtained by CAP3 software (http://asparagin.cenargen.embrapa.br/cgi-bin/phph/cap3.pl), and the identities were compared with the sequences in GenBank using the BLAST program (http://blast.ncbi.nlm.nih.gov/Blast.cgi). The identity matrix was created in the BioEdit program with the alignment and phylogenetic tree developed by the MEGA7: Molecular Evolutionary Genetics Analysis version 7.0 for bigger datasets (Kumar et al., 2016).

### Scanning electron microscopy (SEM)

After isolation, one isolate of each genotype (Hardjobovis and Hardjoprajitno) was subcultered in the three culture media formulations at 28°C for 7 days. Next, the cultures were centrifuged for 5 min at 2,000 rpm, resuspended in 100 μL of fixative (2.5% glutaraldehyde and 2% paraformaldehyde in 0.1 M sodium cacodylate buffer, pH 7.0) and transferred to 24-well polystyrene microtiter plates (Nunc, Roskilde, Denmark) with glass coverslips pre-coated with a thin layer of poly-L-lysine (Sigma Chemical Co, USA). After 1 h, the volume was adjusted to 500 μL of fixing solution to avoid cells adhering to the coverslips, and incubated at 25°C for 12 h. Samples were post-fixed in 1% OsO_4_ (Electron Microscopy Sciences, Washington, PA, USA) and dehydrated in an ethanol series (30, 50, 70, 90, and 100°GL). Samples were critical-point dried with CO_2_ (BALTEC CPD 030 Critical Point Dryer), coated with gold (BALTEC SDC 050 Sputter Coater), and observed under a SEM (FEI Quanta 200, Netherlands).

## Results

The combined formulations of EMJH liquid culture media allowed the growth of *Leptospira* serovar Hardjo strains from three farms (one strain of each farm). Posteriorly, these three strains were characterized by molecular methods as *L. interrogans* genotype Hardjoprajitno (strain Londrina 53) and *L. borgpetersenii* genotype Hardjobovis (strains Londrina 49 and Londrina 54).

Table 2 shows that at the start of the experiment (12 ± 2 days), the first cells were unexpectedly seen in culture medium A with characteristics and movement similar to leptospires, while no cells were seen in culture media B or C. Thus, subculturing was performed using new tubes with the formulation media A, B, and C (A→A; A→B; A→C). After 21 ± 2 days, only subculture A→C presented leptospire cells, but they were few in number and with signs of suffering. On day 27 (±2), this tube was evaluated again and contained many leptospire cells; therefore, media A, B, and C were subcultered again (A→C→A; A→C→B; A→C→C). On day 34 (±3), there was no increase in cell number in subculture AC compared with subculture A→C→B, but there was an increased number of dead (unmoving) cells. In contrast, subculture A→C→B on day 34 (±3) presented many leptospiral cells and was transferred to new subcultures for medium B and C (A→C→B→B; A→C→B→C).

**Table 2:** Evaluation by dark field microscopy of the cultured *Leptospira* (Londrina 49, Londrina 53, and Londrina 54 strains) isolated from naturally infected bovine urine.

**Evaluation date#**	Culture medium^*^	**Dark field microscopic evaluation**	**Subculture**
0 day	A, B, C with antibiotics	NP	NP
1 day	A, B, C with antibiotics	NP	A, B, C without antibiotics
12 ± 2 day	A	Many cells with characteristics and movement similar to *Leptospira*	A→A A→B A→C
21 ± 2 day	A→C	Few structures with signs of suffering	NP
27 ± 2 day	A→C	Good number of leptospiral cells	A→C→A A→C→B A→C→C
34 ± 3 day	A→C	Stagnation of growth with no moving cells	NP
34 ± 3 day	A→C→B	Many cells similar to *Leptospira*	A→C→B→B A→C→B→C
41 ± 2 day	A→C→B→B	One *Leptospira* cell per field	A→C→B→B→B A→C→B→B→C
41 ± 2 day	A→C→B→C	Many cells similar to *Leptospira*	A→C→B→C→B A→C→B→C→C
48 ± 3 day	A→C→B→C	Good number of leptospiral cells	NP
48 ± 3 day	A→C→B→C→B	Good number of leptospiral cells	A→C→B→C→B→B A→C→B→C→B→C
48 ± 3 day	A→C→B→C→C	Few cells	NP
55 ± 4 day	A→C→B→C→B→B	Optimum growth and adaptation to medium B	A→C→B→C→B→B→B

# On the dark field microscopic evaluation date, all tubes were evaluated, but only the significant data are presented in the table;

* *Numbers corresponding to the culture media designated in Table 1; → symbol represents a subculture for another medium; NP, not performed*.

On day 41 (±2), the subculture A→C→B→C, unexpectedly presented more leptospiral cells per field than the subculture A→C→B→B, and subculturing was performed on both tubes (A→C→B→C→B; A→C→B→C→C and A→C→B→B→B; A→C→B→B→C). On day 48 (±3), the subculture ACBC retained good cell growth as did subculture ACBCB. In contrast, subculture ACBCC had cells with less growth.

At the end of the experiment, all medium B subcultures, particularly A→C→B→C→B→B, presented excellent growth and gradually adapted to the standard routine media used in the laboratory without pyruvate sodium, superoxide dismutase, and fetal bovine serum. The isolated strains were named Londrina 49, Londrina 53, and Londrina 54.

**Figure 1:**
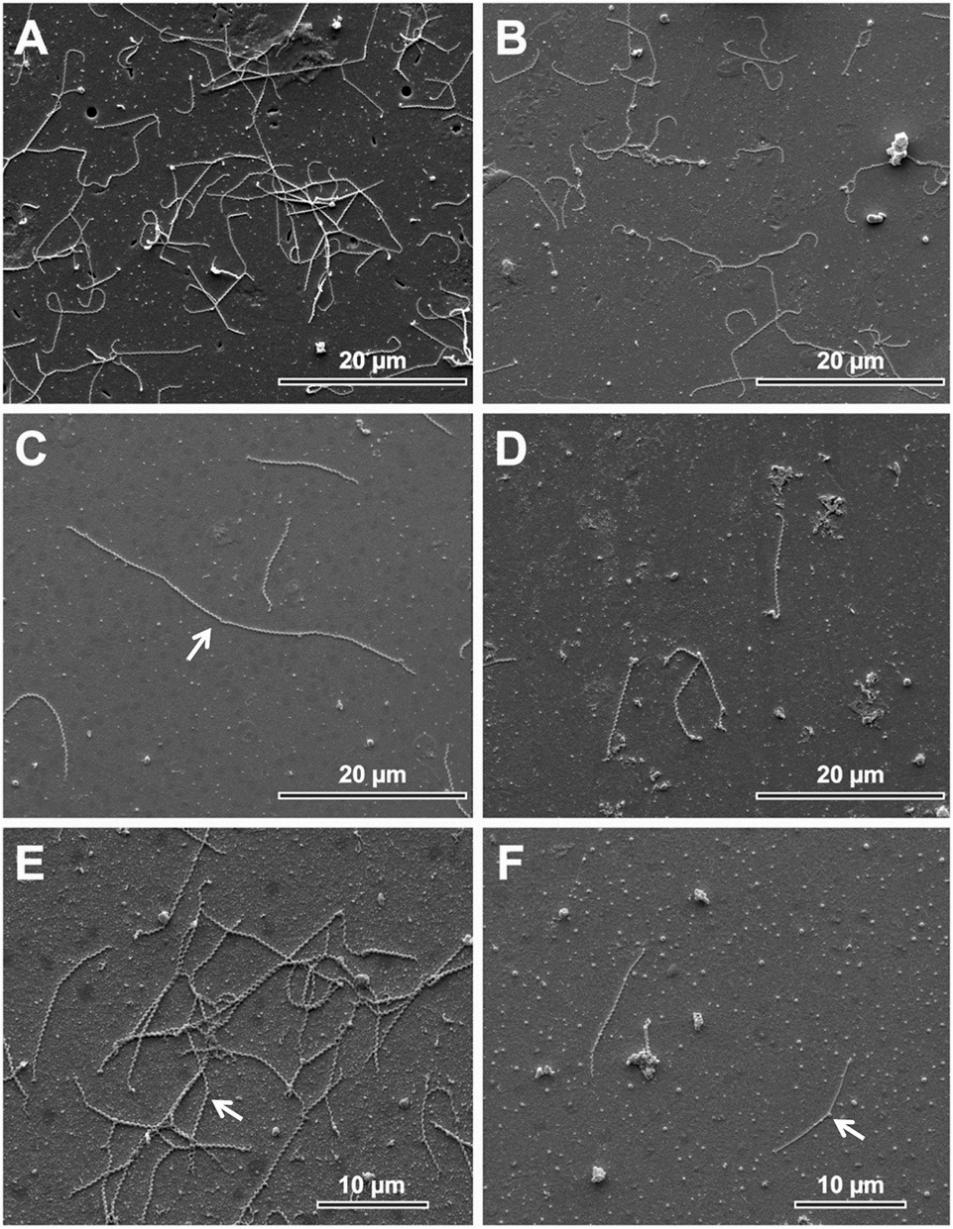
Morphology of leptospire cell in the culture media by scanning electronic microscopy. **(A)** Culture media B with higher number of leptospiral cells of serovar Hardjo; **(B)** Culture media C with fewer number of leptospiral cells of serovar Hardjo; **(C)** culture media B with individualized cells and one elongated cell in division (arrow) of serovar Hardjo. **(D)** Culture media C with fewer cells of *Leptospira* serovar Hardjo in cell division; **(E)** Presence of typical spirochete morphology in culture media B with cells of *Leptospira* serovar Hardjo, Arrow—indicates spirochetal corkscrew morphology; **(F)** Lost of typical spirochete morphology in culture media C with cells of *Leptospira* serovar Hardjo, Arrow—indicates loss of spirochetal corkscrew morphology.

Electron microscopy revealed that the leptospiral cells in culture medium B had a morphology that was more elongated as well as more cells, suggesting more bacterial cell division rate (Figures 1A,C,E). In contrast, there were fewer cells in medium C and those cells had lost the typical spirochete morphology (corkscrew-shaped with hooked ends) (Figures 1B,D,F). Morphologically, medium A was similar to B, but did not have a high number of cells and shown few elongated cells in division (Supplementary Figure 1).

**Figure 2:**
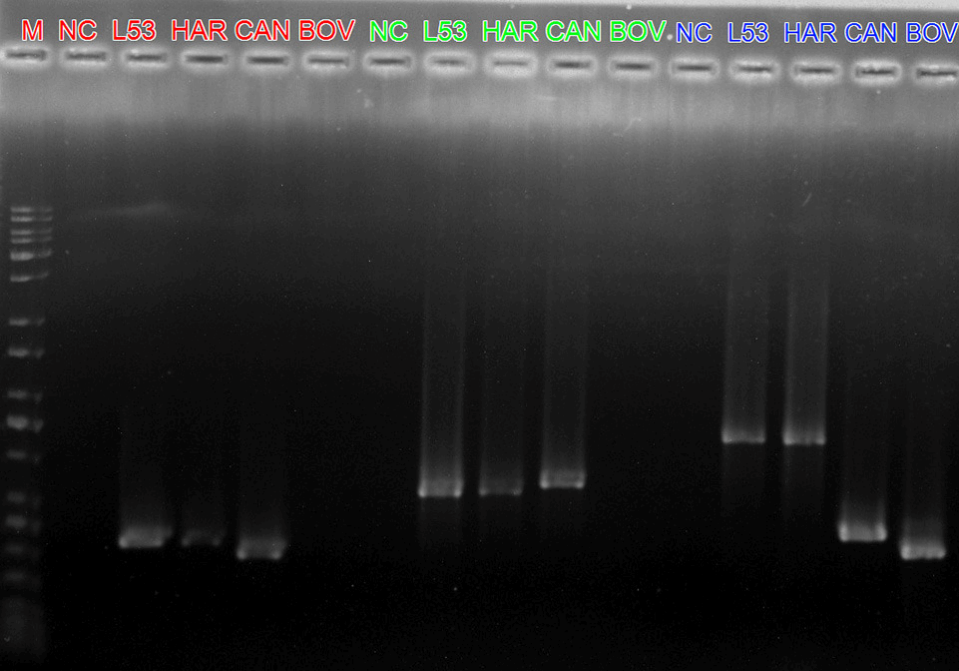
Banding patterns of VNTR visualized in agarose gel. Lane M = 1 kb molecular ladder bp (Kasvi, Curitiba, PR, Brazil), L53 (Londrina 53 strain); HAR = reference sample serovar Hardjo strain Hardjoprajitno; CAN = reference sample serovar Canicola strain Hond Utrecht IV; BOV = reference sample serovar Hardjo strain Hardjobovis; NC = negative control. Locus colors: red (VNTR-4), green (VNTR-7), and blue (VNTR-10).

**Figure 3:**
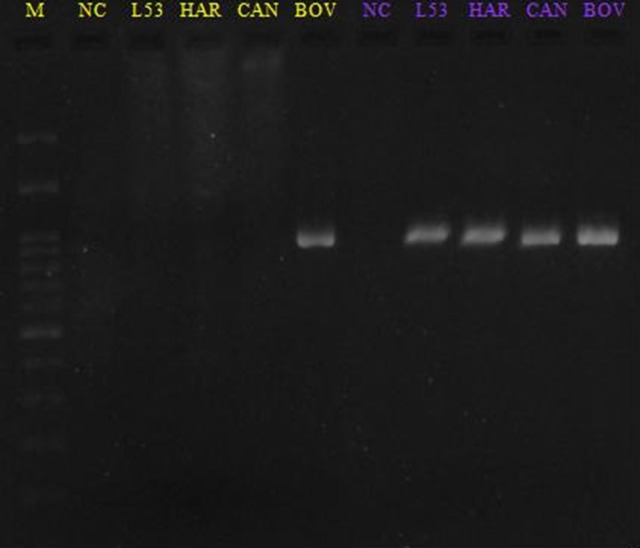
Banding patterns of VNTR visualized in agarose gel. Lane M = molecular ladder bp, L53 (Londrina 53 strain); HAR = reference sample serovar Hardjo strain Hardjoprajitno; CAN = reference sample serovar Canicola strain Hond Utrecht IV; BOV = reference sample serovar Hardjo strain Hardjobovis; NC = negative control. Locus colors: yellow (VNTR-Lb4) and purple (VNTR-Lb5).

The Londrina 53 strain was characterized by MLVA and genetic sequencing, and was identified as *L. interrogans* serovar Hardjo genotype Hardjoprajitno (Figures 2, 3). The other two isolated strains (Londrina 49 and Londrina 54) from the same culture medium formulation were molecularly characterized as *L. borgpetersenni* genotype Hardjobovis, which was previously published (Chideroli et al., 2016).

**Figure 4:**
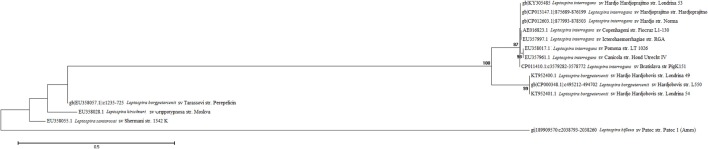
Phylogenetic tree of *Leptospira* with partial *secY* gene sequences of the reference strains and a set of clinical isolates from bovine urine (Londrina 49, Londrina 53, and Londrina 54). The phylogenetic tree was drawn using the maximum likelihood method based on the Tamura 3-parameter model. Reliability of the branches was validated by generating 1,000 “bootstrap” replicates. Evolutionary analyses were conducted in MEGA7. The obtained sequences were deposited in the GenBank database with the following access codes: Londrina 49—KT952400, Londrina 53—KY305485, and Londrina 54 – KT952401.

The phylogenetic tree for all isolates shows that the Londrina 53 strain was grouped in the same cluster as *L. interrogans* and had the sequence identity of the serovar Hardjo genotype Hardjoprajitno. The others strains (Londrina 49 and Londrina 54) remained in the same cluster as *L. borgpetersenii* (Figure 4).

## Discussion

Hardjo is the most prevalent serovar and causative agent of leptospirosis in dairy and beef cattle herds. It causes reproductive failure in livestock worldwide and results in substantial economic loss due to infertility and abortion (Ellis, 2015). In Latin America, few studies have reported recovery of serovar Hardjo (genotype Hardjoprajitno) in cattle (Aycardi et al., 1980; Salgado et al., 2015). In Brazil, two strains, Norma and 2012_OV5, were previously isolated from bovine and ewe, respectively, and characterized as belonging to *L. interrogans* genotype Hardjoprajitno (Cosate et al., 2012; Director et al., 2014). Recently, a Hardjo serovar isolated from bovine urine for the first time in Latin America, was molecularly characterized by MLVA and gene *secY* sequencing as *L. borgpetersenii* genotype Hardjobovis (Chideroli et al., 2016).

EMJH culture media (liquid or semi-solid) is widely used to isolate *Leptospira* (Rodríguez et al., 2002; González et al., 2006; Zacarias et al., 2008; Miraglia et al., 2009; Chakraborty et al., 2011). For many years, our leptospirosis research group has unsuccessfully attempted to isolate serovar Hardjo from bovine urine using unmodified EMJH, even after obtaining positive serology for this serovar (Hashimoto et al., 2017).

In the present study, the combined use of EMJH culture media supplemented with sodium pyruvate (culture medium A); sodium pyruvate and superoxide dismutase (culture medium B); and sodium pyruvate, superoxide dismutase and fetal bovine serum (culture medium C) was used to isolate, adapt, and maintain fastidious leptospires, such as serovar Hardjo, in the laboratory.

The two core ingredients (EMJH medium base and enrichment base) were sustained because they are present in a commonly used medium for *Leptospira* isolation, and sodium pyruvate was included in all culture medium formulations because of a previous report that it enhances *Leptospira* growth when added to a solid medium (Johnson et al., 1973). Superoxide dismutase and fetal bovine serum were chosen to aid the growth of *Leptospira* serovar Hardjo, which has exigent nutritional requirements. Different combinations were made to verify the usefulness of each one. More specifically, superoxide dismutase was chosen because it eliminates the toxic radicals produced during spirochete metabolism that accumulate in the culture medium and the concentration was the same used in the culture media for *Treponema* which is a spirochete as well as leptospires (Austin et al., 1981; Cox et al., 1990). Fetal bovine serum was used to replace the rabbit serum present in the enrichment EMJH base (essential requirement for leptospire growth) because serovar Hardjo is thought to be better adapted to the bovine host. Indeed, future studies using a metabolomics approach could help us to better understand the molecules that are involved in adapting these bacteria to the culture media.

These results show that adding these components to the standard culture medium allowed rapid isolation of the Hardjo serovar. As shown in Table 2, the formulation with only sodium pyruvate (medium A) was where the first *Leptospira* cells were observed and where the primary isolation occurred. For leptospiral culture media, sodium pyruvate has previously been demonstrated to promote growth (Johnson et al., 1973). In a study on hydrogen peroxide damage in mammalian cell cultures, Giandomenico et al. (1997) found that sodium pyruvate was most effective in eliminating hydrogen peroxide and its toxic effects. Therefore, adding this component to the culture medium for isolating fastidious *Leptospira* serovars may be essential for its primary isolation.

After the first simultaneous subcultures with medium B and C (A→B and A→C), fetal bovine serum was found to be critical for initiating growth. However, when it was passed to a new medium with fetal bovine serum (A→C→C) there was no increase in cell growth. In contrast, subculturing the medium with fetal bovine serum to basal medium supplemented with superoxide dismutase without fetal bovine serum (A→C→B→C→B and A→C→B→C→B→B) increased the bacterial growth and final adaptation in the culture medium. In other words, the fetal bovine serum was important for initial growth but eventually, someway became detrimental, and the culture medium required only superoxide dismutase with an enrichment base of EMJH for final leptospiral adaptation. This result suggests that there is a distinct difference between the culture medium requirement for primary isolation and for the maintenance and adaptation of new isolated strains.

More importantly, the change in leptospiral morphological characteristics in the different media suggests that leptospires in the presence of fetal bovine serum will begin to show signs of suffering such as low growth rate, lower replication rate, loss of their corkscrew-shape and loss of their hooked ends, and will eventually die. A genetics study performed with spirochete non-motile mutants indicate that the periplasmic flagella were involved in spirochete motility. It also indicated that the structure of the flagella influenced the shape of the cell ends (Li et al., 2000). Thus, if the culture medium does not provide the elements necessary for bacterial growth and development, or becomes toxic, the cell metabolism and mobility decrease, and thus, lose their hooked ends (Figure 1F). In contrast, the presence of superoxide dismutase seemingly detoxifies the culture medium and allows bacterial development with increased cell size due the possible number of leptospires replicating (Figure 1C).

The ability to isolate and maintain *Leptospira* spp. is critical for both diagnostic and research purposes using molecular characterization to identify new isolates (Adler and de la Peña Moctezuma, 2010). To exchange information between laboratories, the MLVA molecular biology technique is efficient, with easy standardization, rapid clinical diagnosis, and can be applied in the field of epidemiology (Salaün et al., 2006; Slack et al., 2006).

Among the techniques used for molecular characterization of leptospiras, the MLST as the MLVA is a simple PCR based technique. The selected loci of MLST are generally the housekeeping genes, which evolve very slowly over an evolutionary time-scale (Enright and Spratt, 1999). However, this methodology depends of sequencing of seven genes which make the technique more expensive and time consuming. In this study, only the *secY* gene was used, which consists of conserved and variable regions with sufficient sequence heterogeneity to enable the phylogenetic classification of *Leptospira* genus (Victoria et al., 2008; Hamond et al., 2016). Another technique widely used for leptospires genotyping is PFGE, but this methodology requires specific structure/equipaments that may not be available in all diagnosis and research laboratories.

Currently, the MLVA method is one practical alternative for differentiation and identification of the many pathogenic *Leptospira* serovar, including the differentiation of two serovar Hardjo genotypes (Salaün et al., 2006). Furthermore, identifying and typing new isolate strains is important for understanding disease epidemiology in the region, as well as developing diagnostic tools, effective vaccines, and prevention strategies for leptospirosis (Ahmed et al., 2011). The results obtained from the MLVA technique were corroborated by the sequential analysis of the partial *secY* gene, which confirmed the genetic species (Ahmed et al., 2006).

## Conclusion

In this study, culture medium formulations were created to isolate fastidious leptospires of the serovar Hardjo genotypes Hardjobovis and Hardjoprajitno from urine of naturally infected bovine. Noteworthy, additional components were useful for the initial growth (sodium pyruvate and fetal bovine serum) and subsequent maintenance of leptospires (superoxide dismutase) adapted to the medium standard. With this strategy, using three formulations, we succeeded in isolating three pure strains of the serovar Hardjo. After isolation, the technique of MLVA associated with the partial sequencing of gene *secY* have been validated and suggested for molecular characterization of serovars such as Hardjo that may belong to different species. Additionally, an evaluation of leptospire cells in the three formulations by electronic microscopy showed differences in spirochete morphology based on the supplement used in each medium. The superoxide dismutase enzyme induced stretching and cell division; in contrast, cells in the fetal bovine serum medium were fewer in number and lost their corkscrew-shape and hooked ends. Finally, the culture strategies described in this study allowed the isolation and rapid molecular characterization of two serovar Hardjo genotypes inducing new insight into seroepidemiology, specific genotypes, and immunoprophylaxis for leptospirosis in dairy and beef cattle herds.

## Author contributions

JdF, UP, and RC planned the project and designed the experiments. RC conducted the experiments and carried out the data analysis with help from JdF, UP, AFA, DG, and AAA. RC, SS, DG, AdO, and UP contributed reagents preparation and samples collection. RC wrote the manuscript, which was critically reviewed by JdF, AAA, AFA, and UP.

### Conflict of interest statement

The authors declare that the research was conducted in the absence of any commercial or financial relationships that could be construed as a potential conflict of interest.
