# Supplementary figures and images for: Culture Strategies for Isolation of Fastidious Leptospira Serovar Hardjo and Molecular Differentiation of Genotypes Hardjobovis and Hardjoprajitno
Source: Front Microbiol. 2017 Nov 2;8:2155. doi: 10.3389/fmicb.2017.02155 (PMC5673650; doi:10.3389/fmicb.2017.02155)

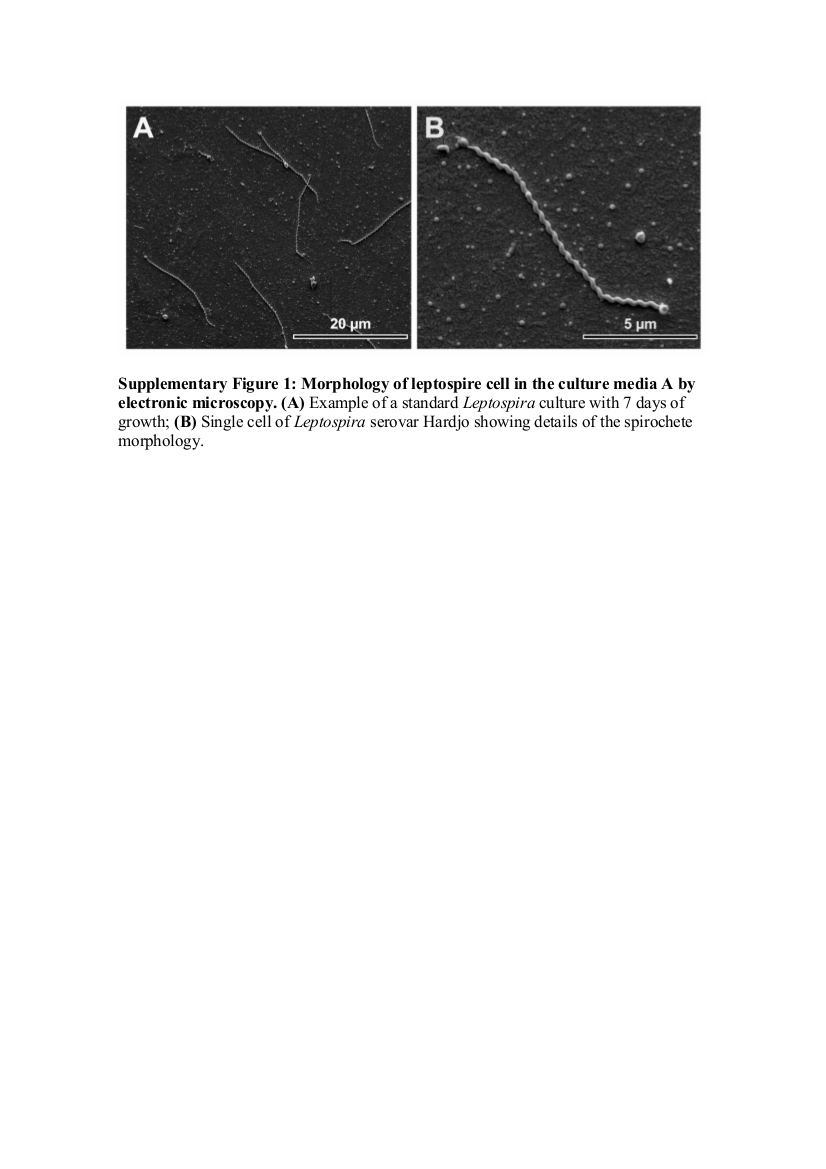

Supplement: Supplementary file 1 [file Image1.TIF]
